# Tool-Condition Diagnosis Model with Shock-Sharpening Algorithm for Drilling Process

**DOI:** 10.3390/s22051975

**Published:** 2022-03-03

**Authors:** Byeonghui Park, Yoonjae Lee, Myeonghwan Yeo, Haemi Lee, Changbeom Joo, Changwoo Lee

**Affiliations:** 1 Department of Mechanical Design and Production Engineering, Konkuk University, Seoul 05030, Korea; bhpark@konkuk.ac.kr (B.P.); dldbswp913@konkuk.ac.kr (Y.L.); audwkd130@konkuk.ac.kr (M.Y.); dlkgoal@konkuk.ac.kr (H.L.); 2 Department of Mechanical Engineering, Stevens Institute of Technology, 1 Castle Pointe Terrace, Hoboken, NJ 07030, USA; cjoo@stevens.edu; 3 Department of Mechanical and Aerospace Engineering, Konkuk University, Seoul 05030, Korea

**Keywords:** tool condition, fault diagnosis system, sharpening algorithm, manufacturing, feature variable, overestimation method, support vector machine, kurtosis

## Abstract

Fault diagnosis systems are used to improve the productivity and reduce the costs of the manufacturing process. However, the feature variables in existing systems are extracted based on the classification performance of the final model, thereby limiting their applications to models with different conditions. This paper proposes an algorithm to improve the characteristics of feature variables by considering the cutting conditions. Regardless of the frequency band, the noise of the measurement data was reduced through an oversampling method, setting a window length through a cutter sampling frequency, and improving its sensitivity to shock signal. An experiment was subsequently performed to confirm the performance of the model. Using normal and wear tools on AI7075 and SM45C, the diagnosis accuracies were 97.1% and 95.6%, respectively, with a reduction of 85% and 83%, respectively, in the time required to develop a diagnosis model. Therefore, the proposed algorithm reduced the model computation time and developed a model with high accuracy by enhancing the characteristics of the feature variable. The results of this study can contribute significantly to the establishment of a high-precision monitoring system for various processing processes.

## 1. Introduction

A fault diagnosis system aims to improve the productivity of a process and reduce the costs by monitoring the manufacturing process and notifying it of any failures in advance [1,2,3,4,5,6]. Machining is one of the processing methods used in product manufacturing. During machining, the tool wears out due to cutting resistance and chips, which affects the surface roughness and dimensional accuracy of a workpiece [7,8]. Several studies have been conducted to diagnose this issue in advance, since tool wear is directly related to the production cost of the product. A diagnostic model was developed to predict tool wear by measuring the acceleration of the main shaft, acoustic signal, load of the main shaft, and cutting load [9,10,11,12]. Totis predicted tool wear by installing a dynamometer on the table of machine tools and monitoring the cutting force during machining [13]. Kalvoda diagnosed the tool condition by applying the Hillbert transform to the acceleration during machining [14]. Li [15] predicted the tool state using the temperature change that occurs during processing, by manufacturing a thermocouple attached to the insert. Jeong measured the wear amount of a drill bit using an optic fiber technique, and diagnosed the wear bit with only simple signal processing [16].

A study has been conducted on a diagnosis model based on machine learning to diagnose the tool wear in real-time. Krishnakumar developed a learning-based tool-condition diagnosis model using a wavelet feature, based on the phenomenon in which the vibration energy generated during processing appears as noise [17]. Patra implemented the thrust force, cutting speed, spindle speed, and transfer speed parameters as feature variables in the drilling process to estimate the number of holes able to be drilled based on tool wear [18]. Caggiano collected process data by installing an accelerometer, a force sensor, and an acoustic emission sensor in a tool holder during the turning process. A model based on pattern recognition was then developed by extracting feature data from the collected data [19]. Ranjan predicted the hole quality of micro drilling, which corresponds to the tool state through a diagnostic model that was developed using the neuro-fuzzy inference system (ANFIS) following data collection, using a tool dynamometer and accelerometer [20]. Wu developed a tool diagnosis model for high-temperature alloy processes by photographing the amount of tool wear during the face-milling process using a charge-coupled device (CCD) [21]. Previous studies have reported a feature-variable-based diagnostic model, which was developed based on the data measured using several sensors during machining. However, signal processing is important because the feature variables are affected by the noise of raw data and the periodicity of the signal. Additionally, the developed model is constructed by extracting feature data using a fixed window length. It does not consider the number of tool revolutions and tool cuttings during the machining process. Each feature variable has a physical meaning and varies based on the characteristics of the signal. Therefore, the characteristics of the feature variable can be improved through signal processing of the raw data.

In this study, the performance of the diagnosis model was enhanced by improving the characteristics of the feature variable in aperiodic signals generated during machining. This method is primarily based on the reduction in the noise of measurement data, regardless of the frequency band, by using an oversampling method that presents the noise reduction effect; it also considers the tool shape information through the cutter pass frequency of the machining process for aperiodic signals. Additionally, the characteristics of the feature variable were improved by calculating a jerk, which improves the sensitivity of the aperiodic impact signal. A diagnosis model was developed to validate the proposed algorithm in the drilling process by collecting the vibration data between the workpiece and the tool, and configuring the feature variables as learning data using the proposed algorithm. The accuracy and computation time are estimated to demonstrate the performance of the proposed algorithm in the drilling process.

## 2. Theoretical Background

Plastic deformation is observed when the cutting tool processes a work piece material, and deformation energy such as noise is generated. Therefore, the work piece is processed under a stable-zone condition with a small dynamic vibration size during processing. In the processing stage, the processing conditions are changed to a non-stable zone due to friction between the tool and the workpiece, movement of atoms between the tool and the workpiece/chip material, thermal stress due to periodic changes in temperature, and mechanical impact [22]. Typically, chips generated during the processing of a workpiece using a cutting tool in the processing stage are discharged or form a build-up edge (BUE) [23]. The remaining chips during the BUE formation process are cured through thermal deformation and plastic deformation. When a chip is attached to the tool surface, it becomes hard and causes collision between the cutting tool and the workpiece [24,25]. The collision between the tool and the workpiece is confirmed as a non-periodical impact signal in vibration, which can be analyzed through statistics [26].

The kurtosis, which is represented in Equation (1), is calculated using standard deviation and the expected value within the section. x, $\bar{x}$, N, and S denote the data, average of data, sample number, and standard deviation, respectively. The kurtosis of standard normal distribution is less than 3. The vibration in the normal condition is stable when the data follows normal distribution. Conversely, kurtosis is more than 3 when the data follows abnormal distribution. When a fault is generated in the system, kurtosis is sharply increased because the tail of distribution follows abnormal distribution. Therefore, a wear tool can be diagnosed by kurtosis, which is changed by an aperiodic shock signal in the time interval [27,28].

(1) $$Kurtosis=\frac{1}{N}\frac{\sum_{i=1}^{N}{\left(x_{i}-\bar{x}\right)}^{4}}{s^{4}}$$

**Figure 1:**
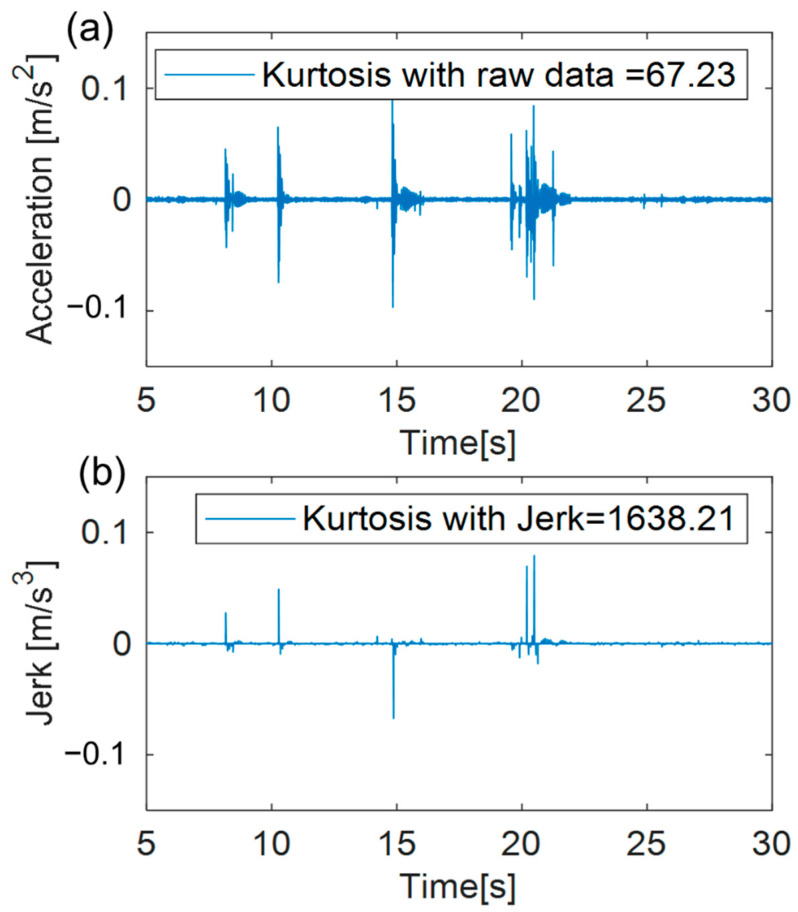
Kurtosis value: (**a**) raw vibration data with aperiodic impulse signals; (**b**) jerk of vibration data with aperiodic impulse signals.

The time interval in the machining process includes the cutter pass frequency and the spindle revolution per minute (RPM). The cutter pass frequency is a frequency at which the shape change of the tool blade can be observed from the measured acceleration data, and is calculated by considering the number of spindle rotations and the number of cutters [14]. The spindle acceleration data confirmed that the frequency of the peak data was theoretically close to the cutting pass frequency. The rotation frequency of the tool whose shape was changed could be confirmed by the cutter pass frequency [29]. Accordingly, the tool shape information is considered when the wear tool analyzes the acceleration signal as the cutter pass frequency. The shock signal is a peak signal that occurs within a short time interval. The rate of change in acceleration is represented as a jerk, whose influence is summarized in Figure 1, corresponding to kurtosis for the signals that contain impact signals. As a result of checking, the kurtosis value for the existing data was 67.23 and the kurtosis value for the jerk was 1638.21. Thereby, when the jerk represents an impact signal in the existing acceleration data and considers it as a kurtosis value, it is more extensively verified based on the impact signal.

## 3. Proposed Algorithm

**Figure 2:**
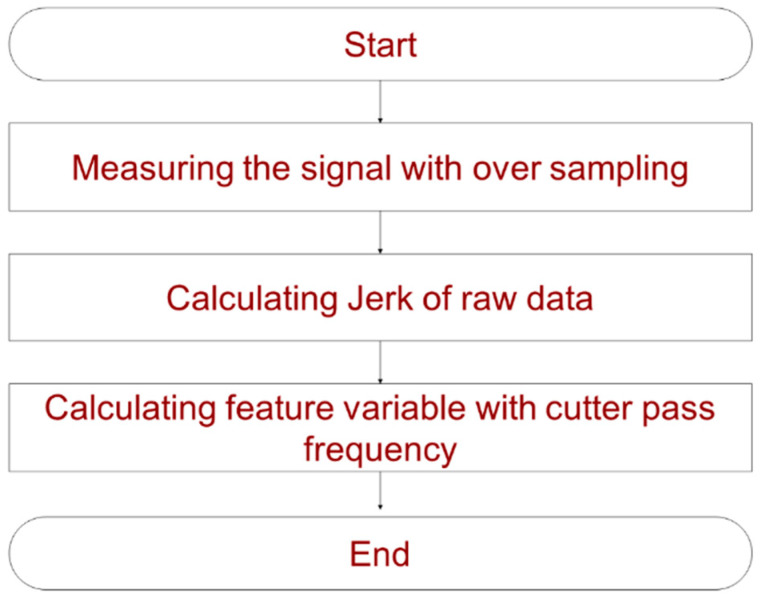
Proposed algorithm for feature variables.

In this study, an algorithm used for extracting aperiodic shock signals during the machining process was proposed. Figure 2 presents the signal processing stage from the signal measurement. The acceleration data with aperiodic shock signals is measured by oversampling, and is then converted into jerk. For the obtained jerk, the feature variables are calculated at cutter sampling frequency through the correlation between the flute of the tool, the main spine rpm, and the sampling frequency.

### 3.1. Oversampling Technique

The noise included in the signal becomes a factor which deteriorates the characteristics when extracted as feature data. A filter may be designed in consideration of a frequency band to reduce the noise included in the signal. However, the filter design requires signal analysis through FFT to obtain the frequency corresponding to the noise [30]. The oversampling can be applied in the signal measurement stage to improve the signal-to-noise ratio (SNR) and signal resolution [31]. The oversampling method sets a frequency higher than the Nyquist sampling frequency, and can improve the signal-to-noise ratio by reducing the noise within the bandwidth of the signal.

### 3.2. Jerk

Jerk represents the rate of change in the unit time of the acceleration signal, and is highly sensitive to the impulse data [32,33]. The aperiodic shock of a single time signal can be clearly observed, as shown in Figure 1.

### 3.3. Cutter Sampling Frequency

The window length of the diagnosis model is a factor that determines the performance of the diagnosis model. Thus, it must be set in connection with the cutting process. Since the signal measures the impact signal generated when the tool blade collides with the workpiece in the cutting process, data sampling is required to consider the rotation cycle of the tool cutter. The cutter sampling frequency (*Fc*) can be defined for one rotation of the tool cutter as follows:

(2) $$Fc=\frac{60Fs}{NR},$$

where *Fs*, *N*, and *R* are the sampling frequency, number of tool cutters, and spindle revolution per minute (rpm), respectively. The period of the feature variable is calculated by the cutter sampling frequency.

## 4. Support Vector Machine

**Figure 3:**
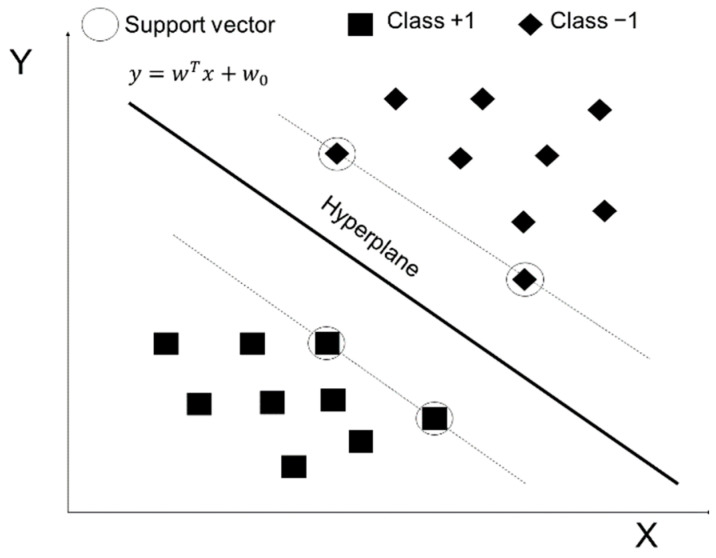
Schematic of the support vector machine.

A fault classification model was developed using learning methods. The support vector machine is an algorithm used to classify data by considering their characteristics [34]. This method is suitable for classification, forecasting, and estimation in small-sample cases. Figure 3 presents an example of data classification using a support vector machine.

The two data clusters were classified using a hyperplane (defined in Equation (3)) formed in the middle of the outer line of one cluster and the adjacent outer line of another cluster. The distance between the hyperplane and datum close to the hyperplane in the outer line of the cluster (named “margin”) was calculated for each cluster. The margin with the maximum distance was obtained to increase the accuracy of the classification of the support vector machine. The two clusters can be classified as +1 and −1 by using Equation (4).

(3) $$y_{i}=w^{T}x+w_{0},$$

(4) $$\{\begin{array}{cc} y_{i}=+1 & x\in C_{1}\\ y_{i}=-1 & x\in C_{2} \end{array},$$

where *w^T^* represents a vector that is normal to the hyperplane, *w*_0_ denotes a scalar, and *x* denotes the input vector in the training data.

If the two clusters cannot be classified using a linear hyperplane, the data can be classified by transferring the two clusters from the input dimension to a higher-dimensional space using kernel functions, which include a polynomial kernel, linear kernel, and Gaussian radial basis functions. The classes of the actual data can be classified into several categories. The class for condition diagnosis of the actual tool should be divided into two levels, since the wear of the machining tool in an actual manufacturing process can affect the quality of a workpiece.

The multiple classes in a support vector machine can be classified using the one-against-all (OAA) and one-against-one (OAO) methods. The OAA method constructs *k* support vector machine models for *k* classes and, the OAO method constructs a support vector machine from two pairs of classes and builds *k*(*k* − 1)/2 models. The OAA constructs a decision boundary for each cluster. Although it has a fast model learning speed, its accuracy is low because an empty space cannot be expressed in the decision boundary. Conversely, the OAO method classifies each cluster into binary, resulting in a higher accuracy and slower learning rate than that of OAA. In this study, we employed the OAA method to construct a model with high accuracy.

## 5. Experiment

**Figure 4:**
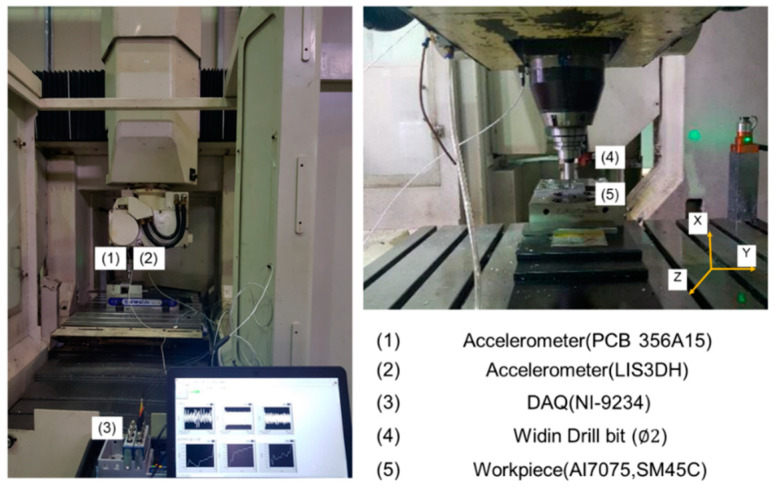
Machining experiment set up.

An experiment was performed using a five-axis machining center (SIC 80/5) to verify the performance of the algorithm, as shown in Figure 4. An acceleration sensor (PCB356A15) was attached to the spindle of the five-axis machining center to measure the vibration signal generated during the machining process. The data were gathered using the LabVIEW DAQ system (NI-9234). The sampling frequency was set to 25,600 Hz based on the oversampling method. A normal and wear ϕ2 carbide tool (Widin SSDL050) was used for the experiment. The machining conditions are listed in Table 1. The minimum quantity lubrication is the process of applying minute amounts of high-quality lubricant directly to the cutting zone used.

**Figure 5:**
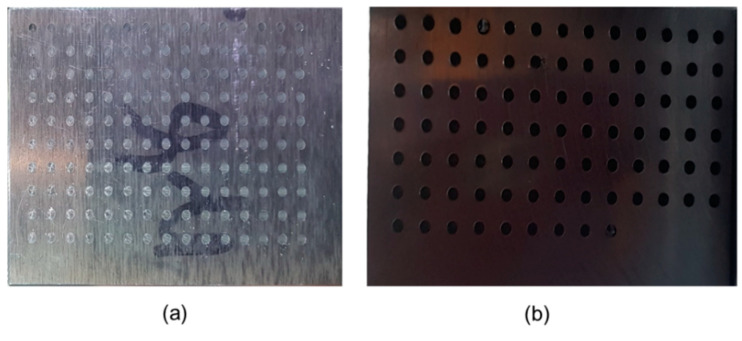
Machined workpieces (**a**) AI7075 and (**b**) SM45C.

The elemental composition of workpiece AI7075 was AI (87.1–91.4), Mg (2.1–2.9), Si (≤0.40), Cu (1.2–2.0), Cr (0.18–0.28), Zn (5.1–6.1), Fe (≤0.50), Ti (≤0.20), Cu (1.2–2.0), and Mn (≤0.30). The elemental composition of workpiece SM45C was C (0.42–0.48), Si (0.15–0.35), Mn (0.60–0.90), P (≤0.030), S (≤0.035), Ni (≤0.20), and Cr (≤0.20). The drill holes had a depth of 10 mm. A peck drilling (with a 2-mm step size) was implemented with minimum lubrication. The machined workpieces, AI7075 and SM45C, are illustrated in Figure 5a and Figure 5b, respectively.

The coordinate system of the sensor was installed based on the spindle. The z-direction is the thrust direction, and the x and y axes are in the radial direction. The proposed algorithm was applied to the z-axis since the z-axis is an important direction during the hole machining process, owing to the thrust force.

**Figure 6:**
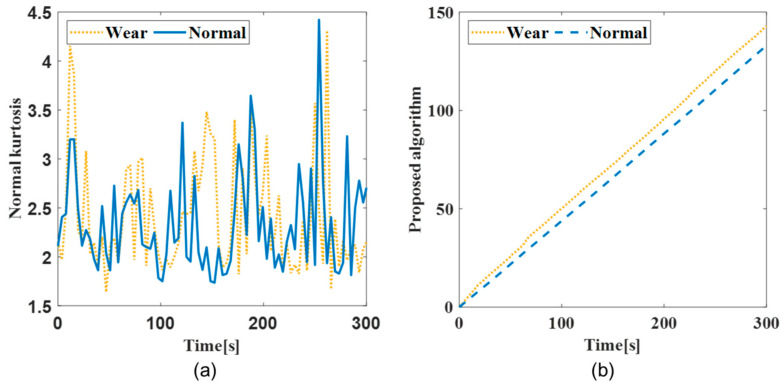
Difference between normal and wear tool in AI7075 machining process: (**a**) kurtosis; (**b**) kurtosis using proposed algorithm.

**Figure 7:**
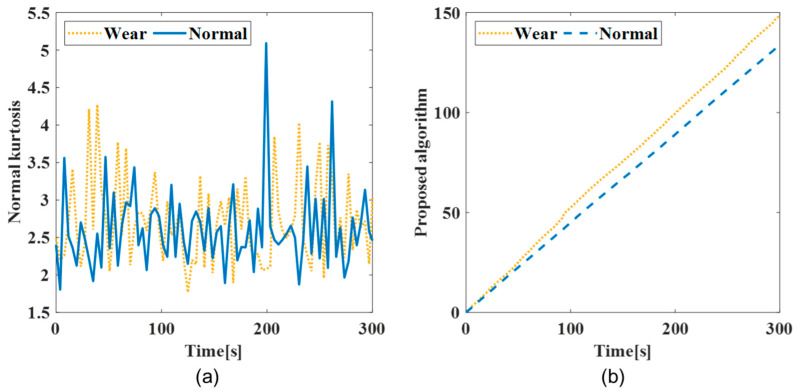
Difference between normal and wear tool in SM45C machining process: (**a**) kurtosis; (**b**) kurtosis using proposed algorithm.

The jerk was calculated for the acceleration data measured by the oversampling method. The window length was confirmed based on Equations (1) and (2), respectively. The window length for the kurtosis was calculated as 153 based on the total of the tool flutes (2), a sampling frequency of 25,600 Hz, and spindle revolution of 5000 rpm. The kurtosis for each workpiece was calculated with the window length. Figure 6 and Figure 7 present the performance of the obtained kurtosis using the proposed algorithm from the vibration data.

Figure 6a shows a kurtosis graph for the AI7075 workpiece, and Figure 7a depicts a kurtosis graph for the SM45C workpiece. Although there was a minimal difference between the data for the normal and wear tools during the initial processing, the sensitivity of the kurtosis for shock signal was verified by using the algorithm proposed in Figure 6b and Figure 7b, to confirm the separation of the normal and wear tools. The difference in the kurtosis was confirmed over time using the proposed algorithm, which was applied to each *z*-axis acceleration datum of the workpiece. The kurtosis graphs for the normal and wear tools were confirmed for each workpiece at the same time. The wear tool exhibited a poor bulk-up edge and chip discharge as machining progressed, and an increased vibration size, resulting in damage to the tool. The slope of the kurtosis instantaneously increased with the increase in the vibration magnitude when an impact signal was generated. It was confirmed that peak vibration was observed in the wear tool. The tool-state graph using kurtosis demonstrated the aperiodic peak vibration of the wear tool.

**Figure 8:**
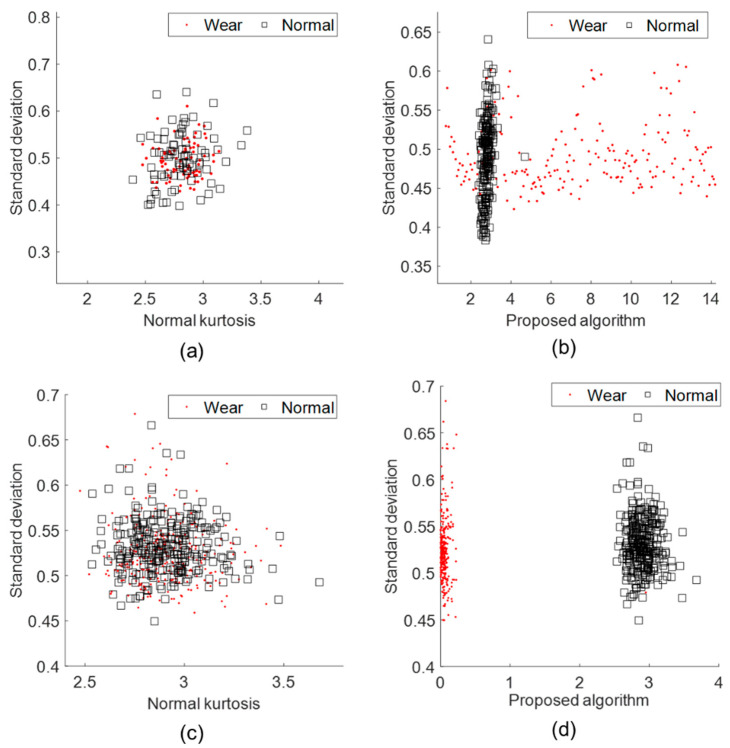
Data Distribution: (**a**) feature variables on AI7075; (**b**) feature variables with proposed algorithm on AI7075; (**c**) feature variables on SM45C; (**d**) feature variables with proposed algorithm on SM45C.

The boundary between the data distribution of the normal and wear tools was confirmed from the comparison of the kurtosis and standard deviation as a scatter plot, as shown in Figure 8. It represents the distribution of the vibration data before the development of a support vector machine-based diagnosis model. The separation criteria of the feature variables must be identified to ensure the accuracy of the machine learning-based diagnostic model. Figure 8a,c confirmed the separability of the data using feature variables. Figure 8b,d confirmed the separability of the data using the feature variables with the proposed algorithm. Generally, high accuracy was expected when constructing a machine learning-based diagnosis model, because the boundary between the data distribution of the normal and wear tools was classified by the calculated feature variable using the proposed algorithm.

The magnitude of the vibration generated when a wear tool collides with a workpiece is larger due to the deformation of the cutting blade. Additionally, it is difficult for the wear tool to discharge the chip, resulting in more residue attached to the surface of the workpiece and generation of a shock signal during machining. The proposed algorithm was used to calculate the feature variable of the impact signal with higher sensitivity than that of the other calculation methods.

**Figure 9:**
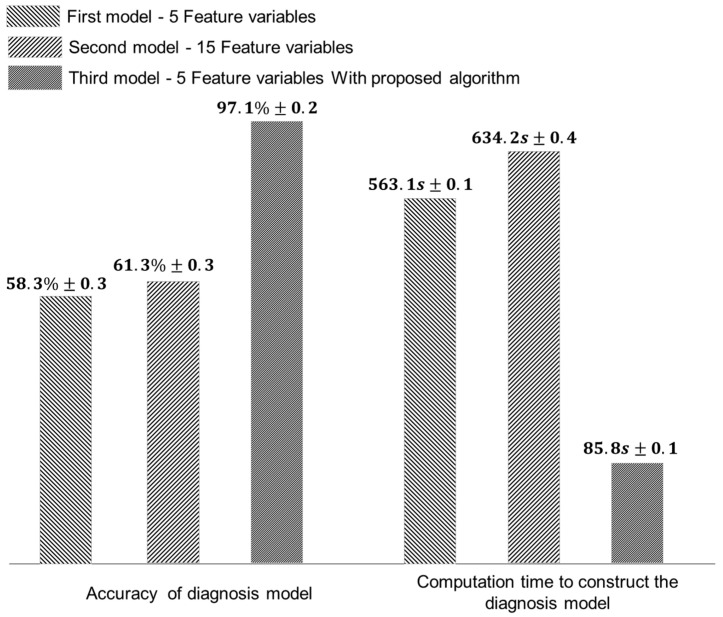
Accuracy and computation time of diagnosis models for AI7075.

**Figure 10:**
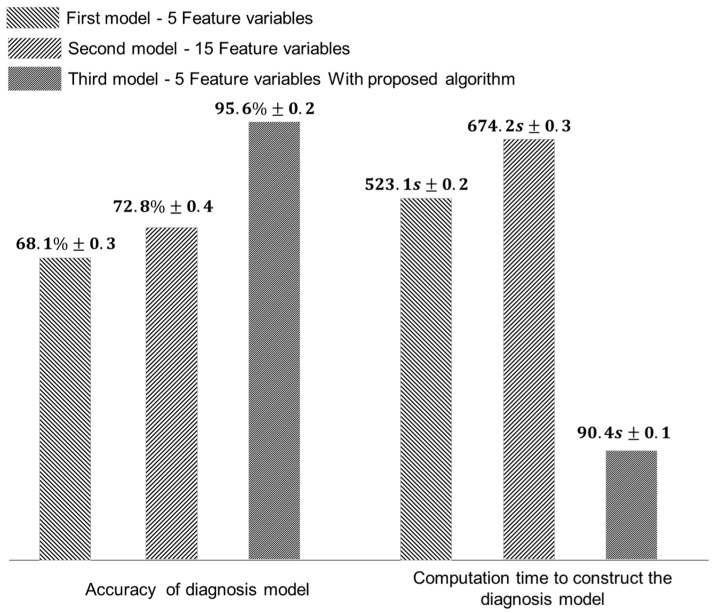
Accuracy and computation time of diagnosis models for SM45C.

A diagnostic model was developed using a support vector machine based on the data measured during the processing of the AI7075 workpiece. A Gaussian distribution was used for the kernel functions to include the nonlinear boundaries. The feature variable used in this process included kurtosis, skewness, standard deviation, root-mean-square (RMS), mean, absolute mean, amplitude of rms, peak-to-peak, peak, form, margin, pulse, standard deviation frequency, center frequency, and rms frequency [35,36,37] (Appendix A). The diagnosis models were developed into three types. The first model was composed of five feature variables including kurtosis, skewness, standard deviation, mean, and root-mean-square. The second model was composed of 15 feature variables, and the third model implemented the proposed algorithm with five feature variables. Figure 9 depicts the performance of each model for the AI7075 machining data. The accuracy slightly increased with the increase in the number of feature variables due to the dimensionality, but it was confirmed that this accuracy was 3% higher than first model. The accuracy of the model increased by 38.8% using the proposed algorithm. Additionally, the computation time required to develop the model was 85.8 s, which was 6.56 times faster than that of the first model. Similarly, Figure 10 shows the performance of the diagnosis model for the SM45C machining data. The second model achieved an increased accuracy of 72.8% when compared to the first model. The accuracy of the third model was 95.6% and the computation time was 90.4 s, which is 5.78 times faster than first model. The diagnosis model for the machining process can achieve high accuracy by increasing the characteristics of the feature variable and by considering the information of the tool shape.

## 6. Conclusions

Machining uses several tools and processing conditions to produce a single product. As the pattern of the vibration data varies depending on the tool and processing conditions, a data-based tool-condition diagnosis model that considers different conditions should be developed. In detail, the performance of the diagnostic model can be improved by screening the valid feature data.

In this study, a shock-sharpening algorithm was developed to improve the characteristics of the feature variables by considering different cutting conditions. The performance of the diagnosis model was confirmed through experimentation. The accuracy of the diagnosis model for normal and wear tools on AI7075 and SM45C were achieved, with an accuracy of 97.1% and 95.6%, and a reduction in computation time of 85% and 83%, respectively. Therefore, the proposed algorithm reduced the model learning time and developed a model with high accuracy by enhancing the aperiodic shock signals to improve the characteristics of the feature variable. Furthermore, the characteristics of the signals were observed by increasing the sensitivity to the impact signals.

The data pattern varied based on the cutting condition, even with a similar material of the tool to the workpiece during processing. Therefore, the results of the proposed algorithm can contribute to the development of a monitoring system with high precision for various processes.

## Figures and Tables

**Table 1:** Cutting condition.

Feed Rate	Cutting Speed	RPM	Depth of Cut	Oil Condition
0.15 mm/rev	31.4 m/min	5000 rev/min	10 mm	MQL

## Data Availability

Not applicable.

## Appendix A

**Table:**

Feature	Equation	Feature	Equation
Mean	$t_{1}=\frac{1}{N_{s}}\overset{N_{s}}{\underset{i=1}{\sum }}x_{i}$	Skewness	$t_{7}=\frac{\left(\sum_{i=1}^{N_{S}}{\left(x_{i}-T_{1}\right)}^{3}\right)}{T_{6}^{3}N_{S}}$
Root-mean-square (RMS)	$t_{2}=\sqrt{\frac{1}{N_{s}}\overset{N_{s}}{\underset{i=1}{\sum }}{\left(x_{i}\right)}^{2}}$	Kurtosis	$t_{8}=\frac{\left(\sum_{i=1}^{N_{S}}{\left(x_{i}-T_{1}\right)}^{4}\right)}{T_{6}^{4}N_{S}}$
Absolute mean	$t_{3}=\frac{1}{N_{s}}\overset{N_{S}}{\underset{i=1}{\sum }}\left|x_{i}\right|$	Form	$t_{9}=f_{2}/f_{3}$
Amplitude of RMS	$t_{4}={\left[\frac{1}{N_{S}}\overset{N_{S}}{\underset{i=1}{\sum }}\sqrt{\left|x_{i}\right|}\right]}^{2}$	Peak	$t_{10}=\max\left(x_{i}\right)/f_{2}$
Peak-to-peak	$t_{5}=\max\left(x_{i}\right)-\min\left(x_{i}\right)$	Margin	$t_{11}=\max\left(x_{i}\right)/f_{4}$
Standard deviation	$t_{6}=\sqrt{\frac{1}{N_{S}}\overset{N_{S}}{\underset{i=1}{\sum }}{\left(x_{i}-T_{1}\right)}^{2}}$	Pulse	$t_{12}=\max\left(x_{i}\right)/f_{3}$
Center frequency (CF)	$p_{1}=\frac{\left(\sum_{j=1}^{k}\left(f_{j}\times p_{j}\right)\right)}{\sum_{j=1}^{K}p_{j}}$	Standard deviation frequency (STDF)	$p_{3}=\sqrt{\frac{\left(\sum_{j=1}^{K}{\left(f_{j}-p_{1}\right)}^{2}\times s_{j}\right)}{\sum_{j=1}^{K}s_{j}}}$
Root mean square frequency (RMSF)	$p_{2}=\sqrt{\frac{\left(\sum_{j=1}^{k}\left(f_{j}^{2}\times p_{j}\right)\right)}{\sum_{j=1}^{K}p_{j}}\;}$		

$x_{i}$ is a signal series of a dataset for $j=1,2,\ldots ,N_{S}$; *N_S_* is the number of data points; $S_{j}$ is a spectrum for j=1,2,…K; *K* is the number of j-th spectrum lines.
